# Distribution and Genetic Characterizations of *Cryptosporidium* spp. in Pre-Weaned Dairy Calves in Northeastern China’s Heilongjiang Province

**DOI:** 10.1371/journal.pone.0054857

**Published:** 2013-01-25

**Authors:** Weizhe Zhang, Rongjun Wang, Fengkun Yang, Longxian Zhang, Jianping Cao, Xiaoli Zhang, Hong Ling, Aiqin Liu, Yujuan Shen

**Affiliations:** 1 Department of Parasitology, Harbin Medical University, Harbin, Heilongjiang, China; 2 College of Animal Science and Veterinary Medicine, Henan Agricultural University, Zhengzhou, Henan, China; 3 National Institute of Parasitic Diseases, Chinese Center for Disease Control and Prevention, Key Laboratory of Parasite and Vector Biology, Ministry of Health, WHO Collaborating Centre for Malaria, Schistosomiasis and Filariasis, Shanghai, China; Obihiro University of Agriculture and Veterinary Medicine, United States of America

## Abstract

**Background:**

*Cryptosporidium* spp. are common parasites of humans and animals. Farm animals, especially pre-weaned calves, are considered to be one of main animal reservoir hosts of *Cryptosporidium* in the transmission of human cryptosporidiosis. The aim of this study was to determine the distribution and genotypes of *Cryptosporidium spp*. in pre-weaned calves using molecular tools and to assess zoonotic transmission and elucidate the public health significance in northeastern China.

**Methodology/Principal Findings:**

A total of 151 fecal specimens from pre-weaned calves were collected in Heilongjiang Province and were screened for *Cryptosporidium* by PCR. The average prevalence of *Cryptosporidium* was 47.68% (72/151). *Cryptosporidium* spp. were characterized by DNA sequencing of the small subunit (SSU) rRNA gene and the 60-kDa glycoprotein (gp60) gene. Based on the SSU rRNA gene, five *Cryptosporidium* spp. were identified, including *C. bovis* (n = 34), *C. andersoni* (n = 26), *C. ryanae* (n = 5), *C. meleagridis* (n = 5) and *C. parvum* (n = 2). The SSU rRNA nucleotide sequences were identical to each other, respectively, within *C. ryanae*, *C. parvum*, *C. meleagridis* and *C. andersoni*. Four types of *C. bovis* were found in the SSU rRNA gene, with two novel types. The gp60 gene was successfully sequenced in one *C. parvum* isolate and three *C. meleagridis* isolates, with IIdA19G1 for *C. parvum* and IIIeA22G2R1 for *C. meleagridis*.

**Conclusion/Significance:**

Molecular analysis indicates that *Cryptosporidium spp*. are endemic in pre-weaned calves in Heilongjiang Province. The findings of *C. parvum* and *C. meleagridis* suggested the possibility of zoonotic transmission and public health significance. The transmission dynamics of *C. parvum* and *C. meleagridis* needed to be clarified by further molecular epidemiologic studies from humans and animals. Whether calves could act as the natural reservoirs of *C. meleagridis* needed to be confirmed by more systematic experimental infection studies.

## Introduction

*Cryptosporidium* spp. are the important intestinal pathogens with a wide host range, having the ability to infect humans and animals including mammals, birds, reptiles, amphibians and fish. Among the susceptible animals, cattle are considered to be one of main animal reservoir hosts of *Cryptosporidium*. Bovine cryptosporidiosis is of great concern due to the huge number of cattle and their economic importance. *Cryptosporidium* infections frequently result in morbidity, weight loss and delayed growth, and sometimes mortality of young animals. Contamination of cattle manure has led to several foodborne and waterborne outbreaks of human cryptosporidiosis [1], [2].

Within the genus *Cryptosporidium*, extensive genetic variation has been reported. To date, at least 23 *Cryptosporidium* species and more than 70 genotypes have been recognized, with new genotypes being found by molecular bio-techniques [3], [4]. PCR-based molecular analysis techniques have identified seven *Cryptosporidium* species in cattle, including *C. parvum*, *C. bovis*, *C. andersoni*, *C. ryanae*, *C. felis*, *C. hominis*, and *C. suis*, and two genotypes *C. suis*-like genotype and the *Cryptosporidium* pig genotype II [5]. The first four *Cryptosporidium* species listed above are responsible for most cases of bovine cryptosporidiosis with different clinical manifestations and there is an age-associated distribution in cattle. *C. parvum* is mostly found in pre-weaned calves with frequent diarrhea; *C. andersoni* is mostly identified in asymptomatic adults, but *C. andersoni*-infected cows have less milk production than uninfected ones. *C. bovis* and *C. ryanae* are frequently found in post-weaned calves and yearlings. Cattle infected with *C. bovis* and *C. ryanae* as well as other *Cryptosporidium* species/genotypes are generally considered to have no visible clinical signs, and no information of subclinical pathology is available. However, there is an exception in cattle in Sweden, with *C. ryanae* in one diarrheal case and *C. bovis* in four cases [6].

Prevalence data have certificated the occurrence of cryptosporidiosis in cattle worldwide and the zoonotic *C. parvum* most commonly identified in pre-weaned calves. In parts of the USA, Belgium, Ireland, Germany, Malaysia, the UK, Sweden, Japan, Spain, Czech Republic and Iran, *C. parvum* is responsible for the majority of *Cryptosporidium* infections in pre-weaned calves and the minority in post-weaned calves and heifers [6], [7]–[17]. However, there appeared to be geographical differences in the population and the preference of *Cryptosporidium* spp. in pre-weaned calves. In some countries or areas, *C. bovis* has been reported to be the most prevalent species in calves [5], [6], [18]–[20].

In China, molecular identification and genetic characterization of *Cryptosporidium spp*. in cattle have been limited to small numbers of *Cryptosporidium* isolates [5], [19], [21]–[23]. Based on previous evidence that pre-weaned calves are the most important source of zoonotic *Cryptosporidium* infection, the present study focused on the investigation of *Cryptosporidium* in pre-weaned calves by PCR and sequencing to better understand the prevalence of *Cryptosporidium* species in pre-weaned calves in Heilongjiang Province. The aim was to examine the distribution and species/genotypes of *Cryptosporidium* in pre-weaned calves at genotype and subtype levels in northeastern China and to assess zoonotic transmission and elucidate the public health significance.

## Materials and Methods

### Ethics Statement

Before beginning work on this study, we contacted the farm owners and obtained their permission to have their animals involved. During specimen collection, all animal work followed guidelines in accordance with the Regulations for the Administration of Affairs Concerning Experimental Animals, and approved by the Animal Ethical Committee of Harbin Medical University.

### Fecal Specimen Collection

Between October 2009 and September 2011, a total of 151 fecal specimens (approximate 10 g each) were randomly collected from six dairy cattle farms in four areas of Heilongjiang Province (Harbin, Daqing, Mudanjiang and Qiqihar). Some were collected directly from the rectum of the animals, while the others were taken immediately from fresh feces deposited on the ground after animal defecation. Their ages ranged from 24 to 60 days. The number of collected specimens accounted for 10–15% of total pre-weaned calves in each farm. All the specimens were stored in 2.5% potassium dichromate at 4°C prior to being used in molecular biologic characterizations.

### DNA Extraction

Potassium dichromate was washed off fecal specimens with distilled water by centrifugation at 1500 g for 10 minutes at room temperature four times. Genomic DNA was extracted from 200 mg of each specimen using a QIAamp DNA Mini Stool Kit (Qiagen, Hilden, Germany) according to the manufacturer-recommended procedures. Eluted DNA was stored at −20°C until further use in PCR analysis.

### Genotying and Subtyping of *Cryptosporidium*

All DNA preparations were screened for the presence of *Cryptosporidium* by nested PCR amplification of an approximate 830 bp fragment of the SSU rRNA gene as previously described [24]. DNA preparations characterized as *C. parvum* or *C. Meleagridis* were subsequently analyzed to determine subtypes using a nested PCR to amplify an approximate 830 bp fragment of the gp60 gene as described [25].

### DNA Sequence Analysis

Purified PCR products were directly sequenced with secondary PCR primers on an ABI PRISM^∧^TM 3730 XL DNA Analyzer (i.e. superscript the TM) by Sinogeno- max Biotechnology Co. Ltd. (Beijing, China), using the Big Dye Terminator v3.1 Cycle Sequencing Kit (Applied Biosystems, USA). Sequence accuracy was confirmed by two-directional sequencing and by sequencing a new PCR product if necessary. The identity of *Cryptosporidium* spp. and genotype was established by comparing the obtained sequences with reference sequences from GenBank by using Clustal×1.83. The representative nucleotide sequences obtained in the present study were deposited in the GenBank database under the following accession numbers: JX416362 to JX416368 (SSU rRNA), and JX416369 and JX416370 (gp60).

## Results

### Prevalence of Cryptosporidium

A total of 151 fecal specimens were screened for the presence of *Cryptosporidium* using PCR amplification of the SSU rRNA gene. 72 specimens were positive for *Cryptosporidium*, thus, the average infection rate was 47.68% (72/151). All the six farms were positive for *Cryptosporidium,* with the infection rate ranging from 7.14% to 100% (Table 1).

**Table 1:** Prevalence and distribution of *Cryptosporidium* spp. in pre-weaned calves in Heilongjiang Province, China by sequence analysis of the SSU rRNA gene.

Area	Farm	No of positive/No of examined (%)	*Cryptosporidium* spp./genotype (no.)
Harbin	Farm 1	16/28 (57.14**)**	*C. bovis* (9); *C. andersoni* (5); *C. ryanae* (2)
	Farm 2	14/23 (60.87)	*C. andersoni* (7); *C. bovis*(7)
Mudanjiang	Farm 3	15/15 (100)	*C. andersoni* (9); *C. bovis*(3); *C. parvum* (2); *C. ryanae* (1)
	Farm 4	2/28 (7.14)	*C. bovis* (2)
Qiqihar	Farm 5	17/44 (38.63)	*C. meleagridis* (5); *C. bovis* (12)
Daqing	Farm 6	8/13 (61.53)	*C. andersoni* (5); *C. bovis* (1); *C. ryanae* (2)
Total		72/151 (47.68)	*C. bovis* (34); *C. andersoni* (26); *C. ryanae* (5); *C. meleagridi*s(5); *C. parvum*(2)

### Distribution and Percentage of *Cryptosporidium* spp

DNA sequencing of the SSU rRNA gene PCR products confirmed the presence of 5 *Cryptosporidium* spp., including 2.78% (2/72) for *C. parvum* on one farm, 47.22% (34/72) for *C. bovis* on six farms, 36.11% (26/72) for *C. andersoni* on four farms, 6.94% (5/72) for *C. ryanae* on three farms, and 6.94% (5/72) for *C. meleagridis* on one farm (Table 2). At least two *Cryptosporidium* species were detected on five farms with the exception of Farm 4 (Table 1). *C. bovis* was the most prevalent and the most widespread in the investigated areas.

**Table 2:** Prevalence and percentage of different *Cryptosporidium* spp. on farms in Heilongjiang Province, China.

Cryptosporidium spp.	No. of Cryptosporidium spp. (%)	No. of areas contaminated	No. of farms contaminated
C. bovis	34 (47.22)	4	6
C. andersoni	26 (36.11)	3	4
C. ryanae	5 (6.94)	3	3
C. meleagridis	5 (6.94)	1	1
C. parvum	2 (2.78)	1	1

### Molecular Characterizations of *Cryptosporidium* spp. at the SSU rRNA Locus

Two SSU rRNA gene sequences of *C. parvum* were identical to each other and showed 100% similarity with the previous cattle-derived sequences (HQ009805, AB513857–AB513881, AB441687, EF611871 and AF093493) and the human-derived sequences (EU331237–EU331241, DQ523504 and DQ656352). All the five *C. meleagridis* isolates had 100% homogeneity with each other at the SSU rRNA locus and were identical to the sequence (HM485432) derived from a British human. 100% homology was observed within the SSU rRNA gene sequences of the five *C. ryanae* isolates, which was identical to those derived from cattle (HQ822137, EU410344, AY587166, AB513679, HQ009807 and HQ179574).

*C. bovis* showed intra-species variations at the SSU rRNA locus in the present study. Sequence analysis of *Cryptosporidium* SSU rRNA gene of 34 *C. bovis* isolates revealed the presence of four distinct types, respectively named as Type I, Type II, Type III and Type IV for a convenient description (Table 3). Type I and Type II had 100% similarity with the cattle-derived isolates HQ179575 and HQ179573, respectively, and Type I was in a high percentage of 91.18% (31/34) of C. *bovis*-positive specimens. Type III and Type IV have never been described previously, with one or two base variations compared to Type I and Type II, respectively (Table 3).

**Table 3:** Intra-species variations in the SSU rRNA nucleotide sequences of *C. bovis*.

Type	No. of isolates (%)	Nucleotide at position*										Accession no. in GenBank
		32	59	276	423	443	447	448	449	453	611	
Ref sequence		A	A	A	C	T	A	T	C	A	G	HQ179575
Type 1	31 (91.18)	A	A	A	C	T	A	T	C	A	G	JX416363
Type 2	1 (2.94)	A	A	G	T	C	G	C	T	G	A	JX416364
Type 3	1 (2.94)	G	T	A	C	T	A	T	C	A	G	JX416365
Type 4	1 (2.94)	A	A	G	T	C	G	C	T	G	G	JX416366

### Subtypes of *C. parvum* and *C. meleagridis* at the gp60 Locus

At the gp60 locus, only one out of the two *C. parvum* isolates produced the expected PCR product and was successfully sequenced. It was identified as the subtype IIdA19G1, which showed 100% homology with the cattle–derived IIdA19G1 isolates (HQ009809 and JF961561) and the human-derived IIdA19G1 isolate (DQ280496), and showed 99% homology with the cattle–derived IIdA19G1 isolate (EF073048) with three base differences. Four out of five *C. meleagridis* isolates produced the expected PCR products, but only three of them were successfully sequenced. All the three *C. meleagridis* isolates belonged to the subtype IIIeA22G2R1, which was not identical to any known *C.* *meleagridis* subtypes.

## Discussion

The prevalence of bovine cryptosporidiosis varies between and within countries in the world. Although hosts of all ages are affected, the prevalence of *Cryptosporidium* declined with the increasing ages of animals. In the present study, the prevalence of *Cryptosporidium* in pre-weaned calves was much higher (47.7%) than a previous study in Henan Province (21.5%), China [5]. It might be attributable to different detection methods, with PCR in this study and Sheather’s sugar flotation technique in Henan [5]. Not surprisingly, molecular methods have the advantage of increased sensitivity of detection. In addition, the differences in prevalence might partially be related to the experimental design, specimen size and host health status at the time of sampling. On the other hand, geographical and seasonal differences can also affect the prevalence of *Cryptosporidium*.

Sequence analysis of *Cryptosporidium* at the SSU rRNA locus indentified five *Cryptosporidium* species in 72 specimens, with 2.78% prevalence for *C. parvum*, 47.22% for *C. bovis*, 36.11% for *C. andersoni*, 6.94% for *C. ryanae*, and 6.94% prevalence for *C. meleagridi*s. *C. bovis* and *C. andersoni* were the two most common species in calves in the investigated areas instead of *C. parvum*. Previously, age-specific preferences for some *Cryptosporidium* species have been observed in cattle. Most *Cryptosporidium* infections in pre-weaned calves are caused by *C. parvum*, whereas *Cryptosporidium* infections in post-weaned calves are mostly caused by *C. bovis* and *C. ryanae*. Two studies in the USA indicated that *C. parvum* is responsible for approximate 85–97% of the *Cryptosporidium* infections in pre-weaned calves [26], [27]. However, recently, there are three studies reporting *C. bovis* as the most prevalent species of *Cryptosporidium* in pre-weaned calves, which respectively occurred in Sweden (74%, 54/73) and in Henan (37.8%, 65/172) and Shanghai (80%, 4/5) of China [5], [6], [19]. In India, *C. bovis* was also observed as the most common species found in both pre- and post-weaned calves [19]. The true reason for the high occurrence of *C. bovis* in pre-weaned calves remains unclear. But early in 2007, Feng et al suggested that the low infection rates of *C. bovis* and *C. ryana*e in calves may be because *C. parvum* may outcompete *C. bovis* and *C. ryana*e [19]. Even in concurrent infection cases with mixed *Cryptosporidium* species/genotypes, unless genotype-specific PCR tool are used, only the predominant species/genotypes will be amplified due to the inherent nature of exponential amplification by PCR [19], [28], [29].

The age pattern of cattle of *C. ryanae* is very similar to that of *C. bovis*. Most *Cryptosporidium* infections in post-weaned calves are attributable to *C. ryanae* and *C. bovis*, which can also be frequently detected in yearlings and adult cattle. The dominance of *C. ryanae* has been proved widely in post-weaned calves although *C. ryanae* is the least prevalent species among the four common *Cryptosporidium* species in cattle [30]. However, some studies also described the presence of *C. ryanae* in pre-weaned calves [13], [19], [20], [26], [27], [31], [32]. Surprisingly, *C. ryanae* was the only species identified in zebu cattle and water buffaloes in a recent study in Nepal [29]. Comparison of the SSU rRNA gene sequences obtained with those from earlier studies identified a nucleotide substitution unique to all *C. ryanae* isolates from Nepal, in addition to some sequence heterogeneity among different copies of the gene [30]. However, the source and meaning of the sequence polymorphism of *C. ryanae* remains unclear. In the present study, five *C. ryanae* isolates (6.94%) were detected out of 72 PCR-positive specimens, with all the *C. ryanae* isolates having 100% homology with each other at the SSU rRNA locus. The findings might be related to the geographical differences in *C. ryanae* distribution.

*C. andersoni* are more frequently found in yearlings and adults [19], [26], [27], [33], but occasionally found in calves, even in pre-weaned calves [10], [12], [27], [32], [34]. In a recent study, *C. andersoni* was the second most prevalent species in pre-weaned calves after *C.parvum* [10]. Similar to the findings above, *C. andersoni* constituted the second largest share (36.11%; 26/72) of positive specimens for *Cryptosporidium* in the present study. In fact, the previous studies clearly demonstrated the susceptibility of pre-weaned calves to *C. andersoni* [10]. However, *C. andersoni* has not been reported to be the most prevalent in pre-weaned calves so far.

*C. meleagridis* has been found in a variety of birds and immunocompromised and healthy humans and recognized as the third most common *Cryptosporidium* species lying behind *C. parvum* and *C. hominis* [35]. In the present study, we obtained a surprising result that *C. meleagridis* was identified in five calf fecal specimens based on the SSU rRNA gene PCR and sequencing. Currently, there are no reports about natural infection of *C. meleagridis* in cattle. Previous experimental cross-transmission studies have showed that *C. meleagridis* has the ability to infect calves. Oocysts of *C. meleagridis*, respectively from chickens and a patient with diarrhea, have been transmitted successfully to calves [36], [37]. Although mechanical transport of *C. meleagridis* in the calves cannot be ruled out, it is likely to be a natural *C. meleagridis* infection in the calves due to the high-intensity oocysts seen in two out of five fecal specimens by microscopy. Whether a calf represents a natural reservoir host for *C. meleagridis* needs to be determined with more systematic experimental infection studies. The transmission dynamics of *C. meleagridis* in calves needs to be elucidated further by characterizing additional *Cryptosporidium* isolates from humans and animals from the same geographic areas at genotype and subtype levels.

By DNA sequence analysis within the partial SSU rRNA gene, intra-species variations were noticed in *C. bovis*. Four types were obtained from 34 *C. bovis* sequences at nine nucleotide sites. Two of them (Type I and Type II) appear to be worldwide with no apparent differences in geographical distribution, while the remaining two (Type III and Type IV) have never been reported previously and might be with characteristic geographical distributions. The findings might represent the genetic characterizations of *C. bovis* in calves in the investigated areas, although the meaning of the sequence polymorphism remains unclear. More data from large-scale investigations regarding the new types of *C. bovis* will contribute to the study of geographical distributions of *Cryptosporidium spp*.

Genetic characterizations of two *C. parvum* isolates in the present study were observed, respectively by analyzing the SSU rRNA gene and the gp60 gene. Within the SSU rRNA gene, both of them showed 100% homology with each other and were identical to those reported in cattle from Egypt, Iran, India, the USA and Henan of China [5], [12], [38]–[40] and in humans from the Czech Republic, Spain and Iran [41]–[43]. Sequence analysis of the gp60 gene showed that the *C. parvum* isolate belonged to the subtype IIdA19G1. The same sequences had been obtained from 67 calves from Henan of China, one calf from Hungary and three HIV-seropositive patients from Portugal [5], [44], [45]. However, the calf-derived IIdA19G1 isolate (EF073048) has three base differences compared to the isolates above mentioned. Due to the lack of epidemiologic data from animals and humans in the investigated areas, we could not draw the definite conclusion about the source and endemic characteristic of C. parvum in pre-calves. The finding of the same sequence of IIdA19G1 identified in Portuguese cryptosporidiosis patients suggested the possible zoonotic transmission of the subtype IIdA19G1 and public health significance [45]. To characterize the transmission dynamics and zoonotic potential of *C. parvum*, numerous studies should focus on subtyping *C. parvum* in farm animals, especially pre-calves. Currently, sequence analysis of the gp60 gene revealed the presence of three subtype families of *C. parvum* in calves worldwide, including IIa, IId and IIl [3], [5]. Subtyping results have shown that calves are commonly infected with the IIa subtype family, and the subtype, IIaA15G2R1, is the most prevalent in most areas studied [3], [46]. IId and IIl subtype families are occasionally found in calves. So far, some IId subtypes have been found in dairy calves in Belgium, Germany, Hungary, Portugal, Malaysia, Spain, Sweden, Egypt, Henan of China, and Belgrade, Serbia and Montenegro [5], [6], [9], [15], [32], [38], [44], [45], [47], [48]. IIl subtypes own the least share among the three subtype families of *C. parvum*. Only 11 isolates presenting five IIl subtypes have been identified in calves, with IIlA16R2 (two isolates) and IIlA18R2 (two isolates) in Slovenia, IIlA24R2 (one isolate) in the Netherlands [49], [50], IIlA16R2 (four isolates) and IIlA17R2 (two isolates) in Belgrade, Serbia and Montenegro [48].

Currently, two subtype families (IIa and IId) have been generally considered to be potentially responsible for zoonotic cryptosporidiosis. It has been confirmed by numerous studies that many of the common IIa subtypes of *C. parvum* in calves have also been identified in humans from the same countries or areas, including North America, Europe and Australia [3]. Within the IIa family, the most common subtype IIaA15G2R1 has been found both in calves and humans in Portugal, Slovenia and the Netherlands [45], [49], [50]. Another subtype IIaA18G3R1 has frequently been found in calves and humans in the same countries or areas, such as Ireland, Northern Ireland and Australia [13], [51]–[55]. Meanwhile, there were four IId subtypes (IIdA17G1, IIdA19G1, IIdA21G1 and IIdA22G1) identified from HIV-infected patients in Lisbon, and two of which (IIdA17G1 and IIdA21G1) were found, respectively in calves and in lambs in the same areas [45]. Human cryptosporidiosis cases in the Netherlands were also reported to be infected with three IId subtypes (IIdA15G1, IIdA16G1, IIdA18G1) of *C. parvum*, which were not found in calves in this study [50]. In a study of genetic classification of *Cryptosporidium* isolates from humans and calves in Slovenia, one IIl subtype (IIlA16R2) has been found in both humans and calves [49]. The finding indicated that IIl subtype families might have the potential of zoonotic transmission.

In the present study, five SSU rRNA gene sequences of *C. meleagridis* were identical to each other and had 100% similarity with the sequence (HM485432) derived from a human case [56]. *C. meleagridis* was found to be as prevalent as *C. parvum* in some countries and areas [46]. It is identified frequently in children and immunocompromised adults from developing and industrialized countries [57]. DNA sequencing of the gp60 gene showed that three *C. meleagridis* isolates sequenced successfully all belonged to the subtype IIIeA22G2R1, and all of them were identical to each other and have never been described previously. Sequence analysis of the gp60 gene has been used widely in subtyping and tracking the transmission of some *Cryptosporidium* spp (*C. meleagridis*, *C. fayeri*, *C. cuniculus*) and genotypes besides *C. parvum* and *C. hominis* [3], [58]–[60]. To date, a total of six different subtype families (IIIa-IIIf) have been identified worldwide [3], [58], [61]. We are unable to determine the true source of infection and transmission dynamics of *C. meleagridis* in calves due to the lack of *C. meleagridis* subtype data from humans and animals in the investigated areas. Even in China, there are no subtype descriptions of *C. meleagridis* although a few studies molecularly identified 22 *C. meleagridis* isolates (seven from wastewater, one from source water, three from chickens, two from quails, three from birds and six from children) [62]–[67].

In conclusion, the present findings, including population, distribution and genetic characterizations of *Cryptosporidium* at genotype and subtype levels in pre-weaned calves, might represent the endemicity of bovine cryptosporidiosis in Heilongjiang Province, China. The reasons behind geographic differences in the distribution of *Cryptosporidium* species/genotypes and subtypes between and within countries remain unclear. Whether the finding of *C. meleagridis* in pre-weaned calves represented a natural infection needs to be confirmed with more systematic characterization of cryptosporidiosis in calves and by experimental infection studies. The transmission dynamics and the risky extent of zoonotic transmission of *C. parvum* and *C. meleagridis* need to be elucidated with sufficient molecular epidemiologic data from humans, animals and water source in the future.
